# Mental Health Self-Tracking Preferences of Young Adults With Depression and Anxiety Not Engaged in Treatment: Qualitative Analysis

**DOI:** 10.2196/48152

**Published:** 2023-10-06

**Authors:** Miranda L Beltzer, Jonah Meyerhoff, Sarah A Popowski, David C Mohr, Rachel Kornfield

**Affiliations:** 1 Center for Behavior Intervention Technologies Department of Preventive Medicine Northwestern University Feinberg School of Medicine Chicago, IL United States

**Keywords:** self-tracking, self-monitoring, self-help, depression, anxiety, young adults, mHealth, technology, qualitative analysis, focus group, personal informatics, mood, thematic analysis

## Abstract

**Background:**

Despite the high prevalence of anxiety and depression among young adults, many do not seek formal treatment. Some may turn to digital mental health tools for support instead, including to self-track moods, behaviors, and other variables related to mental health. Researchers have sought to understand processes and motivations involved in self-tracking, but few have considered the specific needs and preferences of young adults who are not engaged in treatment and who seek to use self-tracking to support mental health.

**Objective:**

This study seeks to assess the types of experiences young adults not engaged in treatment have had with digital self-tracking for mood and other mental health data and to assess how young adults not seeking treatment want to engage in self-tracking to support their mental health.

**Methods:**

We conducted 2 online asynchronous discussion groups with 50 young adults aged 18 years to 25 years who were not engaged in treatment. Participants were recruited after indicating moderate to severe symptoms of depression or anxiety on screening surveys hosted on the website of Mental Health America. Participants who enrolled in the study responded anonymously to discussion prompts on a message board, as well as to each other’s responses, and 3 coders performed a thematic analysis of their responses.

**Results:**

Participants had mixed experiences with self-tracking in the past, including disliking when tracking highlighted unwanted behaviors and discontinuing tracking for a variety of reasons. They had more positive past experiences tracking behaviors and tasks they wanted to increase, using open-ended journaling, and with gamified elements to increase motivation. Participants highlighted several design considerations they wanted self-tracking tools to address, including building self-understanding; organization, reminders, and structure; and simplifying the self-tracking experience. Participants wanted self-tracking to help them identify their feelings and how their feelings related to other variables like sleep, exercise, and events in their lives. Participants also highlighted self-tracking as useful for motivating and supporting basic activities and tasks of daily living during periods of feeling overwhelmed or low mood and providing a sense of accomplishment and stability. Although self-tracking can be burdensome, participants were interested and provided suggestions for simplifying the process.

**Conclusions:**

These young adults not engaged in treatment reported interest in using self-tracking to build self-understanding as a goal in and of itself or as a first step in contemplating and preparing for behavior change or treatment-seeking. Alexithymia, amotivation, and feeling overwhelmed may serve both as barriers to self-tracking and opportunities for self-tracking to help.

## Introduction

Self-tracking is an important element of different therapeutic approaches for psychiatric conditions. In cognitive behavioral therapy (CBT), among the first skills patients learn is how to track thoughts, behaviors, and symptoms [1]. It has been hypothesized that self-tracking serves the following 2 core functions in a therapeutic context: to surface candidate targets for therapeutic intervention more effectively than can be accomplished through periodic clinician-administered assessments and to motivate increases in helpful behaviors while decreasing unhelpful behaviors [2]. For example, self-tracking in the context of CBT is a critical prerequisite skill for cognitive restructuring—or identifying and addressing negative beliefs. Self-monitoring facilitates the identification of negative cognitive biases and enables an individual to address these negative thought patterns through the use of learned skills or approach behaviors (eg, cognitive restructuring, exposure and response prevention). In this manner, self-tracking in the context of therapy can help patients apply skills that, in the long run, may reduce symptoms and promote mental well-being.

In recent years, reflecting the growing ubiquity of mobile and wearable devices, digital tools for self-tracking have proliferated and become popular with consumers. Self-tracking may be coordinated with clinicians or undertaken independently and can include tracking various aspects of one’s context, experience, and behavior (eg, tracking location, step count, moods, finances, and food intake). Many people self-track for behavior change, but they may also self-track out of curiosity and for external rewards [3]. Their self-tracking goals may guide their choice of tool, and self-tracking is often a cyclical process that includes lapses in tracking resulting from forgetting, difficulty maintaining self-tracking, skipping recordings, and suspending self-tracking [3].

Self-tracking has also become a central component of many digital mental health tools and is thought to be relevant for increasing engagement with these tools [4]. A recent systematic review and meta-analysis of approaches to increase engagement in apps for depression and anxiety found that 70% of included apps had a self-monitoring or self-tracking component [5]. This feature is also often incorporated as a component of digital mental health treatments for young people [6]. Digital self-tracking of emotions, symptoms, thoughts, and behaviors that impact mental health can be used to understand mechanistic relationships among symptoms within and across different mental health conditions [7], and self-tracking can provide robust data that are less vulnerable to recall bias in certain conditions like bipolar disorder or depression [8]. Although some digital mental health interventions that include self-tracking simply serve as repositories for self-tracked data, others leverage self-tracking data to deliver personalized interventions or features [9].

Self-tracking can be an attractive feature in digital mental health interventions because it offers the ability for a user to gather data, draw insights about those data, and evaluate potential actions to take as a result of personal tracked data; however, it is not always clear how to leverage these data. Despite being an oft-included feature in digital mental health tools, one recent study of young people and mental health experts found that relatively few young people track mood or other mental health symptoms; however, they were generally interested in using self-tracking data to support their mental health [10]. The authors found that, among other challenges to self-tracking, young people had difficulty knowing what data, if tracked, would support improved mental health management or mental well-being. Additionally, when mental health symptoms were tracked closely, in some scenarios (eg, in the context of eating disorders), it could exacerbate symptoms by increasing attention on dysfunctional symptoms, paradoxically increasing symptoms or distress.

Understanding why people engage in digital mental health self-tracking may be useful for building more effective and engaging digital mental health tools and supporting people on their mental health journeys. For example, a recent study of adult users of mood-tracking apps found that one reason people use mood-tracking is to build insight about the connections among events, contextual circumstances, and subsequent mood [11]. When self-tracking experiences are misaligned to a person’s goals, are inflexible, or are aversive or tracked data do not provide a person with actionable information, people who use digital mental health self-tracking tools may stop engaging in self-tracking [12-14]. Understanding digital mental health self-tracking needs and preferences may be particularly important for young adults, who have some of the highest rates of mental health symptoms but the lowest rates of mental health treatment [15]. Because many young adults are not interested in formal treatment options, digital mental health self-tracking tools may provide an entry into symptom self-management. Unfortunately, to our knowledge, research on mental health self-tracking has not generally focused on the needs and preferences of young people who do not want to engage in formal mental health treatment modalities, in part because of difficulty in engaging these individuals in research [16,17]. These individuals face unique attitudinal and structural barriers to mental health care and form a group who may be able to derive substantial benefit from well-designed digital mental health self-tracking tools. To this end, there remain crucial questions as to the specific mental health self-tracking needs of young people not engaged in treatment.

To better understand the self-tracking needs of young people who were not treatment engaged, we conducted 2 asynchronous online discussion forums to understand (1) the types of experiences young adults not engaged in treatment have had with digital self-tracking for mood and other mental health data and (2) how young adults not engaged in treatment want to engage in self-tracking to support their mental health.

## Methods

### Participants

We recruited 50 young adults (aged 18-25 years) with moderate-to-severe depressive or anxiety symptoms after they voluntarily completed an online screener for depression (Patient Health Questionnaire-9 [18]) or anxiety (Generalized Anxiety Disorder-7 [19]) on the website of Mental Health America, a national mental health patient advocacy group. Alongside their results, people with a score of 10 or higher on either screener were presented with a link to learn more about our study and an eligibility survey. In prior research, this cut point has predicted moderate-to-severe major depressive disorder [18] and generalized anxiety disorder [19], and in clinical contexts, this cut point is often used as the basis for referral to treatment (eg, [20]). Potential participants completed an additional eligibility survey, and they were eligible if they self-reported as being US residents between 18 years and 25 years old (19-25 years in Nebraska because of the state’s legal age of majority) with sufficient English language to participate in the study procedures, willing to use a mobile phone, and not engaged in treatment. Exclusion criteria were visual, voice, hearing, or motor impairments that would prevent them from completing study procedures; self-reported serious mental illness; or suicidal ideation with a plan and intent. Of the 725 individuals who completed the eligibility survey, 106 met the criteria for inclusion. Researchers invited eligible individuals to participate, aiming to include a sample of participants who were diverse in terms of age, gender, race, ethnicity, and symptom severity and who were representative of the users of Mental Health America's screening site. Eligible individuals were invited until the groups were considered full (~25 per discussion group). See Table 1 for the demographic information of the sample.

**Table 1:** Participant demographics of the full sample (N=50) who participated in online asynchronous remote communities about experiences with and interest in digital mental health services between April 2020 and June 2020.

Characteristics		Results	
Age (years), mean (SD)		21.46 (2.23)	
PHQ-9^a^, mean (SD)		15.62 (4.43)	
GAD-7^b^, mean (SD)		14.05 (5.28)	
**Gender, n (%)**			
	Female	38 (76)	
	Male	6 (12)	
	Nonbinary	2 (4)	
	Prefer not to answer/not reported	4 (8)	
**Race, n (%)**			
	White	28 (56)	
	More than one race	6 (12)	
	Asian	5 (10)	
	Black or African American	3 (6)	
	American Indian or Alaska Native	1 (2)	
	Prefer not to answer/not reported	7 (14)	
**Ethnicity, n (%)**			
	Hispanic/Latinx	8 (16)	
	Not Hispanic/Latinx	38 (76)	
	Not reported	4 (8)	

^a^PHQ-9: Patient Health Questionnaire 9.

^b^GAD-7: Generalized Anxiety Disorder 7.

### Procedure

Participants who provided informed consent were enrolled into 1 of 2 online discussion groups based on asynchronous remote community (ARC) methods [21,22]. These groups were run in sequence between April 2020 and June 2020, with the second group following the first to probe deeper about digital mental health experiences and preferences and to gain clarity around questions that were not fully answered in the first group. A group size of approximately 25 each was chosen so that groups would be large enough to produce engagement and represent diverse perspectives while being small enough that participants could read and respond to others’ contributions without becoming overwhelmed. Groups were hosted on the FocusGroupIt website [23]. The ARC methodology was selected to decrease participant burden compared with in-person research and to facilitate dialogue among participants while preserving anonymity [21,22,24]. Researchers posted a new prompt on a message board every 3 days, and participants were asked to respond to all prompts in their ARC group session and to comment on at least one other participant’s response to each prompt. All participant responses were anonymous and visible to all other participants in their ARC group. The first group (n*=*28) included 6 total sessions over 18 days, and the second group (n=22*)* included 8 sessions over 24 days. Prompts were related to participants’ experiences with and interests in digital mental health services. The FocusGroupIt website saved all responses, which researchers exported and used for data analysis. Researchers analyzed participant responses to the subset of prompts that were relevant to self-tracking, which included 1 session from the first ARC group and 4 sessions from the second ARC group (see Multimedia Appendix 1 for prompts analyzed). Each of these sessions had an average of 50 responses in total, contributed by an average of 20.4 participants per session. The average response length was 105.7 words.

### Ethical Considerations

Study procedures were approved by the university’s institutional review board (#STU00211168). All participants completed informed consent procedures.

To mitigate risk in the interactive ARC groups, ARC groups began with a code of conduct established in consultation with a clinical psychologist, groups were monitored by the research team daily for compliance, and resources were provided for 24/7 support, which participants were advised was not the purpose of the group. A risk management protocol was in place, in which any posts signaling elevated suicide risk would prompt the study team to reach out to the participant for further assessment of risk and to connect them to resources, if needed. However, no posts triggered this protocol.

Researchers compensated participants US $8 for their response to each prompt and US $2 for up to one substantive response to another participant’s response to each prompt.

### Data Analysis

We conducted a thematic analysis following the steps outlined by Braun and Clarke [25]. For this study, we analyzed responses to 1 prompt from ARC session 1 and 4 prompts from session 2 based on their relevance to our research questions. First, 1 researcher (MLB) read through all the data. Next, using the qualitative coding software Dedoose, 3 coders (MLB, RK, and SAP) open coded the data session by session, meeting after coding each session to build and refine a shared codebook and to resolve coding differences through discussion. Such a process of “consensus coding” can increase consistency and circumvent individual biases of the researchers, allowing them to arrive at consensus about the themes in the data [26]. Once consensus was reached, such that further discussion did not yield further codebook revisions, coders individually completed coding on the remaining sessions. The coders and another researcher (JM) then met to collate the codes into themes and to check the data against the themes, reducing redundant themes and identifying hierarchical relationships. Example quotes were then selected for the final report.

## Results

### Past Self-Tracking Experiences

Although our study focused on individuals with mental health concerns, participants reported using self-tracking in the past for a wide variety of health, work, and lifestyle-related variables. They reported having had mixed experiences, with negative reactions to calorie and weight tracking and more positive experiences in other domains. Some participants reported past experiences self-tracking mood and mental health, whereas others had never considered it before. For those who had self-tracked their mood in the past, some found it helpful, whereas others reported experiencing barriers shared with other types of self-tracking.

#### Negative Past Self-Tracking Experiences

##### Highlighting Unwanted Behaviors

Participants described how self-tracking made them more aware of unwanted behaviors, particularly with calorie counting and weight tracking apps. Tracking their eating and weight highlighted their dissatisfaction with their eating and weight, which made them feel worse about themselves. As P41 stated:

While I had downloaded the calorie count app as a way to help me start eating healthier or possibly lose weight, it just made me feel horrible to see how unhealthy my eating habits were. In the end, I decided it was just best to uninstall it instead of being hyper-focused on my eating and my weight.

P41

Participants reported discontinuing self-tracking related to food and weight because they found that tracking made them so focused on their eating or weight that they became stressed and their eating behavior became more disordered.

One participant reported a similar negative reaction to self-tracking finances, rather than food and weight:

I always wonder where my money goes and what it is that I do to spend so much of it. I don't like writing it down because it gives me anxiety seeing how much money I have actually spent.

P50

The act of self-tracking brings more awareness to the behavior being tracked, which can be distressing when the person self-tracking is dissatisfied with that behavior or becomes obsessed with changing it.

##### Reasons for Discontinuing Self-Tracking

A common theme was that self-tracking requires effort, and some participants stopped self-tracking in the past either because they lost motivation or simply forgot. Even when participants were very motivated to start tracking and did it frequently for a short time, they reported that, once they missed a few days or started dismissing reminders to tracking, they would eventually stop tracking altogether. P39 reported the following:

I have also gone through the cycle of downloading self-betterment apps, using them for a bit, and then deleting them. It is so difficult to maintain these apps, and so easy to ignore.

P39

Self-tracking requires cognitive effort and remembering, and once motivation wanes, the effort may no longer seem worth it.

Some noted that setting goals that were too ambitious and trying to track data that were difficult to find (eg, nutritional information) contributed to trouble maintaining a self-tracking practice. For some participants, falling short of their behavioral goals, including falling short at self-tracking consistently, was disappointing, frustrating, or overwhelming and led them to discontinue self-tracking. As P33 reported:

I tried those apps also and then I get frustrated because I don’t use it and then feel like I am failing for not tracking my mood. I don't think that helps me at all.

P33

This highlights that self-tracking can lead to self-criticism with regards to the self-tracking practice itself not just for the behaviors being tracked.

Other participants reported discontinuing tracking because it was not helping or no longer seemed necessary (eg, losing motivation to track mood during periods of better mental health). P25 reported the following:

I've tried mental health tracking apps like Sanvello but have lost interest quickly because it takes so much time to record my feelings and seems pointless when I'm doing well.

P25

Motivation to self-track mood can wane during periods of more positive mood, leading to stopping self-tracking. Taken together, we see that participants discontinued the effortful practice of self-tracking when they forgot, saw little benefit or reason to track, or felt bad due to self-tracking.

#### Positive Past Self-Tracking Experiences

##### Tracking Wanted Behaviors and Tasks

In contrast to the aforementioned negative experiences, participants also reported positive self-tracking experiences in the past, particularly when tracking behaviors they wanted to increase, rather than decrease, and when they were tracking discrete things that were clear how to track. Participants reported positive experiences self-tracking their work and school assignments, to-do lists, step counts, menstrual cycles, and bills. When participants reported finding self-tracking helpful, they reflected that tracking helped them feel less overwhelmed or more accomplished or tracking provided clarity needed for behavior change (eg, tracking finances elucidates where to cut back on spending, tracking periods lets participants anticipate timing and prepare). P33 reported the following:

I have found that when I am behind and when I am in need of handling a lot of things and it feels overwhelming, I make a checklist. So, I can feel accomplished when completing each task. It makes it much easier for me to get through hard days. I think I have used checklists more when I am more depressed and anxious. It has helped a lot.

P33

In addition to the feeling of accomplishment, participants reported that keeping track of tasks helped them remember necessary things when they felt disorganized or unfocused and that this sometimes helped them feel better.

##### Tracking and Processing Mood

For constructs that are more open-ended, without clear start and end points, like mood, having a structure to make self-tracking more discrete was helpful for some participants. One participant reported finding a CBT workbook helpful for identifying moods, and others reported using self-tracking apps that had a limited number of icons or categories for different moods and mental health symptoms. However, this type of structure acted as a barrier for another participant, who reported finding it hard to map their experiences onto discrete ratings:

I've tried a few mood tracking apps on my phone where you rate how you feel daily. But I always have a tough time finding a reference point from which to base my ratings. Like, am I really feeling as bad as possible? Will it get worse?

P13

Although some participants reported liking mobile apps that make tracking more automated, discrete, or faster, other participants preferred a more open-ended journaling format, either in an app or analog journal. They described that the process of writing things out helped them process their feelings or improved their mood. However, some of the participants who described liking journaling noted that they discontinued the practice after some time despite the positive experience because of lack of time or motivation. P41 reported the following:

The mood app was the one I used the most just because we got to journal about our day or our feelings, but I never really used the information that it saved about my moods. I just liked the journaling part. However, I eventually stopped using it because I felt I no longer had the time to sit down and journal everything I'd like to put down.

P41

In contrast to the benefits of structure and discrete behaviors noted in the previous section, some participants preferred open-ended journaling, but they also noted drawbacks of being less actionable and requiring more time and effort.

##### Gamification for Motivation

Some participants reported that gamification elements of self-tracking apps were motivating or helpful for them, specifically elements that rewarded progress toward a goal (eg, “leveling up”) or disincentivized unwanted behaviors. P47 reported the following:

There's an app I like called Forest that lets you set up a timer for how long you need to stay away from your phone. If you successfully refrain from using your phone during that time, you'll be able to grow a little tree to plant in a virtual forest. If you fail to do so, however, the tree will die and you won't have anything to grow in your forest.

P47

Another participant mentioned liking making a character. These gamified elements, coupled with reward systems, seemed to enhance motivation, contributing to some participants’ positive past self-tracking experiences.

#### Design Considerations That Young Adults Not Seeking Treatment Want Self-Tracking Tools to Address

When thinking about what they would want in a digital tool to support their self-tracking of mood and other aspects of mental health, participants highlighted 3 major needs they wanted the tool to address. First, they wanted self-tracking to help them understand their feelings better, including patterns in their moods. Second, they wanted self-tracking to provide some organization, motivation, and structure when their lives felt chaotic or overwhelming. Finally, because of the effort involved in self-tracking that led so many participants to discontinue in the past, they wanted a tool that would be simple to use.

#### Building Self-Understanding

##### Identifying Feelings

Some participants reported difficulty identifying their feelings and expressed interest in a tool that could help them better understand these feelings. Identifying what they were feeling could serve as a first step toward understanding why they feel that way and could potentially help them communicate better or face those feelings. P36 suggested they would want to use a tool that could provide the following:

Something more along the lines of learning how to identify feelings. Like an app where you can answer certain questions and it will tell you how you're feeling? For myself, most of the time I can't quite identify what I'm feeling other than ‘bad’ or ‘can't do anything’. I don't know if that would help in any way but at least I wouldn't feel stupid for not knowing how to articulate how I feel.

P36

However, in the next session, P36 added the following concern about identifying feelings in the absence of identifying steps to change them:

Apart from the obvious uncomfortable experience of acknowledging your own negative state, just stating it doesn't really do much of anything (i.e offer a solution). In other words, not doing anything about it is what I feel makes me more upset.

P36

Although identifying feelings may be a benefit of self-tracking, some participants wanted it to be in the service of emotion regulation or behavior change.

##### Self-Tracking Mood in Relation to Other Variables to Gain Insight Into Patterns

A major theme was that participants were very interested in self-tracking mood to understand patterns in their mood like how their mood changes over time, what events trigger changes in mood, and how other behaviors and variables relate to changes in their mood. Participants highlighted how connected their mood was to other things in their lives and tracking some of these other factors in relation to mood could help them have a deeper understanding of themselves.

Participants specified wanting to track sleep, exercise, food, screen time, and events in relation to their mood. P42 reported the following:

I think it could be helpful to have an app where you can track your mood and what happened that day or attach tags of some kind to help you understand patterns and triggers - like for example, if you consistently feel upset after a specific event, like a fight with a family member, you can track that and see those patterns over time.

P42

Others wanted to understand how their mood relates to their thoughts in specific situations, which might require some more open-ended reflection, like journaling prompts or a survey. P7 reported the following:

I've seen a couple of apps that encourage you to journal your reflections on the days and also a rating to the day in the mode of emojis. So, if it was a good day, the emoji would be a happy face, etc. That simple measure of mood would be good to track. Paired with the qualitative data of the reflection journal, it might be possible to analyze and determine the thoughts that are correlated to the emotions felt at the end of the day.

P7

For some participants, building self-understanding was a goal in and of itself; for others, the utility of self-understanding was to see long-term progress that would be difficult to see day to day or to identify ways to make changes. P31 reported the following:

The potential of being able to look back and understand what correlates to your mood swings, for example, could be nice, giving you something to target and find a solution for.

P31

Different types of insights were expected to be gleaned from tracking mood over time, in relation to thoughts, and in relation to other variables, and participants varied in which of these types of self-understanding they hoped to gain from self-tracking.

##### Visualizations for Self-Understanding

Because participants noted that it can be difficult for them to understand complex data about themselves while they are immersed in their everyday lives, participants suggested a variety of visualization types to help make sense of their tracked data to build self-understanding. These included summaries, calendars, charts, and graphs. Participants wanted these visualizations to understand both change in mood over time and change in mood in relation to other variables. They highlighted that these visualizations could be helpful in identifying trends, for example when their mood is mood worsening or improving, and how effective different strategies are in supporting mood regulation in different contexts. P13 reported the following:

I also wish I could sync it with a digital calendar of events, to identify different triggers. Like, do I feel worse after a change in my life? Or do I feel significantly better or worse after doing x or y? I would really appreciate summaries and visualizations of these things, and how they change over time. I'm a sucker for good data and statistics, and that's how I try to understand how my depression affects me.

P13

#### Organization, Reminders, and Structure

##### Self-Tracking to Support Tasks of Living

In addition to building self-understanding, participants described ways of using tracking that were more pragmatic and focused on making progress with day-to-day goals and responsibilities. Participants commented on how difficult it is to keep up with activities of daily living (eg, brushing teeth, grooming, cooking, eating, drinking water, cleaning) when they are feeling depressed and how they would like a self-tracking tool to remind them of these basic activities that are foundational to their lives and motivate them to do these tasks.

Some participants came up with specific design solutions for how self-tracking could support activities of daily living. One suggested gamifying these activities so more complex tasks that come up infrequently are worth more points, whereas completing basic daily activities still earns points (albeit fewer). P44 focused more on the benefits of reminders within a self-tracking system:

I think a lot of people with depression can experience a kind of brain fog, where its just hard to remember what you're doing and what you need to be doing. If there was an app that helped keep you on track with your goals, or even just a reminder to drink some water or brush your teeth, it would be helpful for a lot of people.

P44

Relatedly, participants reflected how difficult it was to stick to a routine or find motivation when depressed, including following through with self-tracking. P37 reported the following:

I have a hard time getting started and being motivated enough to want to do it, and remembering is hard too.

P37

Participants reported that they would like a tool to remind them of their plans and motivate them to follow through, including through reminder notifications, though some participants noted that too many notifications would become annoying, highlighting a tension in this design principle. This desire for reminders extended to reminders to use the self-tracking tool.

##### A Sense of Stability and Accomplishment

Participants highlighted that self-tracking supports their routines and provides a sense of accomplishment, stability, or calm. For some, this comes from an app checking in with them throughout the day, and for others, this comes from taking stock of progress toward a goal. P32 reported the following:

There could also be a section with different goals to achieve, personal or just random, that you can check off as you go. I think it would be useful because checking things off, or completing a whole list of tasks gives a feeling of relief.

P32

Other participants reported that having a plan for things like schoolwork and finances provided this structure and feeling of stability, whereas for some, using self-tracking to clarify routines in sleep or mood provided a sense of control.

#### Simplifying the Self-Tracking Experience

##### Balancing Short and Long Inputs

Given the themes around participants discontinuing self-tracking due to high effort required and their desire for self-understanding, it makes sense that participants reported wanting to be able to give very simple inputs when self-tracking. Some reported disliking typing long responses and thought that selecting a word, number, or emoji from a list of options to represent their mood would make it easier to self-track. Related to prior positive experiences with journaling, some participants also wanted the richness and flexibility of optional journaling prompts, with most suggesting journaling at the end of the day. P30 reported the following:

i think if i could maybe use an app that simply tracks my mood throughout the day by just checking off a mood. That way at the end of the day, I can go back and log my moods with a bit more detail in an actual journal and be able to add whatever I feel I need to.

P30

Participants wanted to make self-tracking as simple as possible with short inputs but retain the benefit of longer inputs as an option when convenient.

##### Including Mood-Regulating Components

Participants also suggested that the convenience of having activities or recommendations for emotion regulation built into the same tool as self-tracking would simplify the experience and motivate them to use the tool. These included uplifting messages or mantras, guided meditation videos, coloring, music, and a way to chat with a provider. P38 reported the following:

I have a few apps that each do one part of this but I love the idea of having one ‘master app’ that combines them all. Would definitely make it easier to stay on top of things and remember to do things if it was all in one space.

P38

Although adding more features might not make a self-tracking tool simpler, it might simplify the process of emotion regulation to have self-tracking and mood-regulating tools in one place.

## Discussion

### Main Findings and Implications

Young adults not engaged in treatment reported that various forms of self-tracking have been and could be beneficial for their mental health and well-being but that self-tracking is often cumbersome, leading them to discontinue. In this discussion, we connect our findings to the broader literature on self-tracking and behavior change in mental health conditions. We also discuss how these findings may be translated into improved digital mental health tools for this population, emphasizing that self-tracking must be made simpler, while addressing needs for self-understanding and structure.

Our findings suggest that experience with self-tracking using digital tools was common for these young adults, including using various self-tracking apps, although tracking had not consistently been explicitly focused on mental health. Participants reported mixed satisfaction with past self-tracking experiences, with negative experiences tracking calories and weight and more positive experiences tracking other behaviors, tasks, and moods. This difference may relate, in part, to whether the user aims to increase or decrease a target behavior via self-tracking. If they are trying to decrease a behavior, tracking may become a punishing experience, bringing awareness to something about which they feel guilt or shame. However, when tracking a behavior they want to increase or if the goal is more insight-oriented or exploratory, each instance of tracking may serve as a reward, highlighting progress toward a goal. Prior work has similarly found that users reported discomfort or guilt with the greater awareness self-tracking brought to unwanted behaviors like calories and spending [27]. Consistent with this research, past work on self-tracking has also observed the potential for tracking to bring out compulsive thinking patterns and behaviors [13]. Although reflecting on positive experiences may improve long-term happiness [28], perseverating on negative experiences may unintentionally lead to rumination [29]. Since individuals with mental health concerns like depression and anxiety are susceptible to rumination and distorted thinking patterns, they may be particularly vulnerable. Although we observed negative self-tracking experiences mainly in the domain of weight and food, mental health conditions often co-occur with body dissatisfaction and eating disorders.

On the other hand, our findings suggest that tracking to advance self-understanding may be of particular value to young adults experiencing depression and anxiety. Participants reported considerable interest in self-tracking as a way to advance self-understanding, including of emotions and how emotions connect to other behaviors and facets of life. This self-understanding objective might be contrasted with using self-tracking to change behavior or ameliorate mental health symptoms, and it is consistent with prior research that found people turn to mood-tracking apps during difficult times in order to increase self-awareness and self-reflection [11]. This focus on self-understanding may reflect that mental health symptoms and states are more subjective than physical health–related variables, which can make self-tracked data more challenging to interpret and act on in service of behavior change [30]. Additionally, these participants were young and likely early in their process of recovery. The transtheoretical model of behavior change (also called the “stages of change” model) posits that particular processes of change align better or worse with individuals depending on where they fall on a spectrum of readiness for change [31]. These participants are likely in the contemplation stage (ie, considering whether they will make a change in how they manage mental health) or preparation stage (ie, making plans to take action in support of recovery), but many have not committed to or carried out a plan of action to improve their mental health [32,33]. In these early stages of change, helpful processes are proposed to include consciousness raising (gaining information and awareness about a problem and its solutions), dramatic relief (getting in touch with one’s emotions and expressing oneself), environmental reevaluation (assessing how the problem affects the physical and social environment), and self-reevaluation (emotional and cognitive reappraisal of one’s values as they relate to the problem) [31,34]. These are arguably all processes that could be supported by a mental health self-tracking tool. Understanding patterns of mood and symptoms over time, including relationships with stressors and other variables, may allow individuals to feel empowered and more in touch with their feelings and behaviors, and this could potentially be a precursor to better managing mood and symptoms as they move through the stages of the behavior change process.

The focus on self-understanding of moods also relates to alexithymia, the experience of being unable to identify or distinguish between one’s emotional states. Alexithymia can be part of the experience of depression and other mental health conditions, and it is often experienced as frustrating and distressing. Although challenges in labeling emotions were observed to make tracking mood difficult (ie, it takes longer and is perceived as more difficult to record moods), participants also saw potential for self-tracking to help them learn to better label their moods over time. Participants talked about the ways they would like to input their mood data, including picking from a list of emotions (or a set of visuals, like emojis), which provides an emotion vocabulary to work with and makes it easier and faster to record data. Although journaling (or entering free text) on its own was rarely the preferred method to track mood, it was seen as a helpful complement to list-based tracking, allowing users to choose to elaborate on why they selected a particular mood at a particular time, the relevant contexts, or any insights they had.

Another key finding of our work was the benefit many individuals perceived in self-tracking that is highly pragmatic (eg, bills, assignments, chores), which had potential to contribute to a sense of well-being through helping participants organize important aspects of their lives. When depressed, people often experience a lack of motivation and difficulty concentrating and remembering things. Necessary tasks can pile up as they struggle to complete basic activities of daily living, like brushing teeth and tidying their space. Similarly, when anxious, people tend to feel overwhelmed and have difficulty prioritizing among many tasks that all feel important. Given these common symptoms, it makes sense that self-tracking the basic tasks that are important in getting through the day and working toward one’s goals would be appealing to people experiencing depression and anxiety. Self-tracking may prevent the pile-up of tasks that only worsens feelings of failure and being overwhelmed, and self-tracking tools may reduce cognitive load and contribute to feelings of control. Similar to the discussion about alexithymia, though, amotivation and difficulty remembering things can serve not only as opportunities for self-tracking to help but also as barriers to self-tracking, as people may forget to self-track. Notifications or reminders to do and track activities can address difficulty remembering, and rewards (either gamified rewards or the feeling of accomplishment from completing tasks) can address amotivation. Also, it is possible that some of the desire for self-tracking basic tasks of living may relate to the developmental stage of the young adult participants in our study, who may be experiencing more adult responsibilities and tasks than they did in their teens.

### Limitations and Future Directions

Although we conducted 2 separate discussion groups, our sample of 50 may not represent the experiences of all young adults not engaged in treatment. We recruited participants through voluntary online screeners, so our sample may be more interested in online tools to understand their mental health than others who would not seek out and complete these screeners. Our sample contained more women than men and nonbinary participants and represented non-Hispanic White identities more than other racial and ethnic groups. Finally, self-reported experiences, needs, and preferences for self-tracking tools may differ from experiences actually using those tools.

This work suggests future directions. We employed a qualitative approach to build understanding of how self-tracking operates in a population of which there is limited understanding: young adults who are not engaged in treatment. Qualitative approaches can be helpful in formative work to characterize a phenomenon and generate insights and testable hypotheses that may be the basis of future work using a quantitative approach [35]. For example, future work may use more structured questions or a survey to examine whether and how participants’ stated levels of interest in different types of tracking predict their actual behaviors. It may also examine the relative interest in and use of self-tracking across a range of behaviors and whether harms and benefits of self-tracking relate to the specific behavior being tracked. Future studies may also explore whether people who hold different identities (eg, race, gender, age) have different experiences with and preferences for self-tracking.

### Design Implications

One of the common reasons participants reported discontinuing self-tracking was the effort or time involved, and participants highlighted that simplifying the experience of self-tracking was important to them. These responses are consistent with prior work on self-tracking, which found that people tend to stop using self-tracking tools that do not fit with their conception of themselves; do not provide sufficiently useful, interesting, or actionable data; and require too much effort or maintenance to fit into their routines [14]. Although many common themes came up in this research, there was considerable variety among participants in some of their specific preferences and reactions to different features, highlighting that creating a simple self-tracking experience may mean tailoring a tool to a person’s goals, preferences, and challenges. For example, some participants reported positive reactions to to-do lists, finding them motivating, while others had negative reactions, noting that they highlighted how little the person had done. These may stem from how participants interpreted the lists, with more negative reactions coming from interpretations like “I can’t do it all” versus more positive interpretations like “I am making progress.” Having a to-do list bounded to a small number of items and providing rewarding feedback may be particularly important for maintaining more positive interpretations.

Another important question that should guide design is whether the person is looking for self-understanding, behavior change support, or structure during a period of feeling overwhelmed. A tool for self-understanding might focus more on tracking emotions or symptoms and related triggers and behaviors, and it might include more space for free-text journaling. Designers should consider how much time the user has and how much time they want to spend on self-tracking. A person with more time might be willing to track more frequently, track more variables, and use longer input types (eg, more free text) than a person with less time. Relatedly, people vary in how they like expressing themselves (eg, through prose, single words, emojis, numbers, colors, or pictures). Tools may offer users a choice of what input type they would like to use, or they may rely primarily on less time-intensive input types like picking from a list of words, emojis, or numbers, with an option to expand on entries with more expressive free text, images, colors, or audio inputs. Designers may consider nudges to reflect differently on positive and negative emotional experiences in order to optimize self-understanding while minimizing the chance that users will fall into rumination. Designers should also consider what symptoms users are experiencing and how those may serve as barriers to self-tracking. People who have difficulty keeping track of things may benefit from more reminders, but reminders should not be so frequent as to annoy someone during a busy period. People may also vary in their preferences for data visualizations to help them make sense of their self-tracked data, especially in light of their varying goals for self-tracking. However, visualizing self-tracked data in ways that are easily interpretable is quite challenging, and understandable visualizations may need to focus on only a subset of the data [36].

### Conclusions

Our study suggests that young adults not engaged in treatment see value in self-tracking tools designed to meet their needs, including to deepen self-understanding, manage disorganization in their lives, and address motivational challenges that can often impair tracking. Digital self-tracking tools that are simple to use and allow young adults to work toward these goals may help prevent worsening of symptoms and meet their needs at their current stage of development and readiness for change.

## Appendix

Multimedia Appendix 1 Asynchronous remote community (ARC) group prompts.

## Abbreviations

ARC: asynchronous remote community
CBT: cognitive behavioral therapy

## Data Availability

The data sets analyzed during this study are not publicly available due to the sensitive nature of the data and the risk of reidentification of participants. Information about the codebook is available from the corresponding author on reasonable request.
