# Supplementary material for: Mental Health Self-Tracking Preferences of Young Adults With Depression and Anxiety Not Engaged in Treatment: Qualitative Analysis
Source: JMIR Form Res. 2023 Oct 6;7:e48152. doi: 10.2196/48152 (PMC10589825; doi:10.2196/48152)
Supplement: Multimedia Appendix 1 [file formative_v7i1e48152_app1.docx]

Multimedia Appendix 1: ARC Group Prompts Analyzed in the Present Study

ARC Group 1, Session 6: Messaging, goal setting, and tracking

We want to get your thoughts on receiving text messages to help when you feel sad, down, or empty. Think back to the last time you were feeling depressed or had a bad day. Please give us some examples or ideas of text messages that would have been helpful to get that day. Give us some examples or ideas of text messages that would have been unhelpful to get that day (or made it worse).

Some technologies allow people to track things going on in their lives, including healthy and unhealthy patterns (e.g., step counts, sleep, eating), how they feel (mood), or their progress toward their goals (checklists). We are interested in what your thoughts are about collecting and using information related to your feelings and mental health. What information would you want collected on your smartphone about your feelings, mood, and anything else about your mental health, and why? How would you want to see that information you've tracked about your mental health on your phone?

**ARC Group 2, Session 2: How Technologies can help**

In the last session we asked about activities you could do to help manage feelings of being sad or down. In this session, we are interested in whether you see any ways technology could help you to carry out those activities.

Imagine you have an unlimited budget to build an app, chatbot, web series, online program, email list, or any other digital tool that could help people like you carry out positive activities. What kind of program would you build, and how would it work?

If you don't think technology could help with self-management of mental health concerns, please tell us why not.

**ARC Group 2, Session 3: Goal setting, and tracking**

Some people find it helpful to track things going on in their lives, including healthy and unhealthy patterns (e.g., step counts, sleep, eating), how they feel (mood), or their progress toward their goals (checklists).

Have you ever tried to track the things going on in your life in order to help with your mood or mental health? What did you track, and how did you make use of that information?

What information (if any) would you want to collect on your smartphone or computer about your feelings, mood, or anything else in your life that relates to your mental health?

**ARC Group 2, Session 5: Messaging Part 2 (Contexts)**

We'd like to know more about your feelings about text messaging. Specifically, imagine that you signed up for a program where you receive automated text messages on your phone several times a day to support your mental health.

When you think about receiving text messages on your phone, are there things going on in your life that would change how receptive you would be to mental health-related text messages, or that might change the type of text messages you would want to receive.

These factors might include things like the time of day, how busy you are, whether you're alone or with others, the activities you're engaged in, your mood, etc. Which of these factors is most important to think about when designing a text messaging program, and why?

**ARC Group 2, Session 7: Making text messages personalized for you**

Thanks for everything you've shared so far. We really appreciate your insights! This will be the last question about technology and messaging.

Even though everyone here has some things in common like your age group and some mental health-related experiences, there are important ways you are different from one another. We are interested in ways we can adapt digital tools for mental health so they work better for you, specifically.

One way to do this is to collect information when a person starts using the tool (like having them fill out a survey or rate different kinds of possible messages). If you were starting to use a text messaging program for mental health, how many questions would you be willing to answer about yourself and your preferences?

So that the messages can address what's going on in your life, what kinds of information would it be important for you to share in order for the messages to work welI for you?

Are there other ways you can envision customizing a digital mental health messaging program (for example, writing your own motivational messages, or changing what topics you were most interested in)?
